# Hostility Modifies the Association between TV Viewing and Cardiometabolic Risk

**DOI:** 10.1155/2014/784594

**Published:** 2014-06-23

**Authors:** Anthony Fabio, Chung-Yu Chen, Kevin H. Kim, Darin Erickson, David R. Jacobs, Janice C. Zgibor, Tammy Chung, Karen A. Matthews, Steven Sidney, Carlos Iribarren, Mark A. Pereira

**Affiliations:** ^1^Department of Epidemiology, Graduate School of Public Health, Epidemiology Data Coordinating Center, University of Pittsburgh, 130 DeSoto Street, 127 Parran Hall, Pittsburgh, PA 15261, USA; ^2^Department of Psychology in Education, School of Education, University of Pittsburgh, Pittsburgh, PA 15261, USA; ^3^Division of Epidemiology and Community Health, School of Public Health, University of Minnesota, Minneapolis, MN 55454, USA; ^4^Departments of Psychiatry and Epidemiology, School of Medicine, Graduate School of Public Health, University of Pittsburgh, Pittsburgh, PA 15261, USA; ^5^Division of Research, Kaiser Permanente Northern California, Oakland, CA, USA

## Abstract

*Background*. It was hypothesized that television viewing is predictive of cardiometabolic risk. Moreover, people with hostile personality type may be more susceptible to TV-induced negative emotions and harmful health habits which increase occurrence of cardiometabolic risk. *Purpose*. The prospective association of TV viewing on cardiometabolic risk was examined along with whether hostile personality trait was a modifier. *Methods*. A total of 3,269 Black and White participants in the coronary artery risk development in young adults (CARDIA) study were assessed from age 23 to age 35. A cross-lagged panel model at exam years 5, 10, 15, and 20, covering 15 years, was used to test whether hours of daily TV viewing predicted cardiometabolic risk, controlling confounding variables. Multiple group analysis of additional cross-lagged panel models stratified by high and low levels of hostility was used to evaluate whether the association was modified by the hostile personality trait. *Results*. The cross-lagged association of TV viewing at years 5 and 15 on clustered cardiometabolic risk score at years 10 and 20 was significant (*B* = 0.058 and 0.051), but not at 10 to 15 years. This association was significant for those with high hostility (*B* = 0.068 for exam years 5 to 10 and 0.057 for exam years 15 to 20) but not low hostility. *Conclusion*. These findings indicate that TV viewing is positively associated with cardiometabolic risk. Further, they indicate that hostility might be a modifier for the association between TV viewing and cardiometabolic risk.

## 1. Introduction

Over the past several decades, television (TV) viewing has emerged as a ubiquitous recreational pastime [1, 2]. Epidemiological evidence supports excess TV viewing as a social/environmental exposure that may increase risk of cardiovascular disease—the leading cause of morbidity and mortality [3, 4]. Associations between excessive TV exposure and cardiometabolic risk are consistently observed in many countries [5–11].

Hostility, a nonmodifiable personality trait, has been reported to predict increased risk of cardiovascular disease through a number of pathways including increases in blood pressure, heart rate, and stress-related hormones [12]. The amount of TV viewing and hostility are highly correlated, and people with high hostility may be more susceptible to TV-induced negative emotions [13]. The contents of TV programs may evoke negative emotional responses and aggressive behaviors, especially among people with a predisposition towards hostility. For instance, hostile viewers react to violent TV news with moral emotions, including anger and contempt [14]. Hostile people also show a propensity toward unfavorable health behaviors including smoking, drinking problems, unhealthy diet, and less physical activity which in turn may be influenced by TV viewing [12]. Thus, the associations between TV viewing and cardiometabolic risk may be stronger for those with high hostility compared to those with low levels of hostility.

It is hypothesized here that the hostile personality trait may be an important effect modifier for the association between TV viewing and cardiometabolic risk, with those with high hostility having a stronger association between TV viewing and cardiometabolic risk. The hypothesis that the direct association between TV viewing and cardiometabolic risk may be modified by a propensity towards a hostile disposition has, to our knowledge, never been examined.

## 2. Methods

### 2.1. Participants

The coronary artery risk development in young adults (CARDIA) study is a prospective study designed to investigate the development and risk factors of cardiovascular disease. At baseline (1985-1986), 5,115 young adults between the ages of 18 and 30 were recruited [15]. The same participants were followed during 1987/8 (year 2), 1990/1 (year 5), 1992/3 (year 7), 1995/6 (year 10), 2000/1 (year 15), and 2005/6 (year 20). Because measurements of TV viewing were collected at year 5, 10, 15, and 20, the present study used data at these four follow-up examinations. Participants were excluded based on these conditions: (1) pregnancy; (2) medication use or history of use for hypertension, hypercholesterolemia, or diabetes at year 5; (3) missing covariate data at year 5.

### 2.2. Measures

#### 2.2.1. Television Viewing

Number of daily TV viewing hours was assessed by a self-administered questionnaire at years 5, 10, 15, and 20. Participants were asked, “during leisure time do you watch television?” and “on average, about how many hours per day do you watch television?”

#### 2.2.2. Cardiometabolic Risk

A continuous clustered cardiometabolic risk score was created according to a metabolic syndrome cluster score and has demonstrated face validity [16]. A similar score has been published by other studies [9, 17, 18]. Each participant was assigned a Z-score for each of the following components: waist circumference, HOMA insulin resistance (fasting glucose × fasting insulin/22.5) (natural log), fasting triglycerides (natural log), HDL-cholesterol, and systolic blood pressure. The Z-scores (z = (value − mean)/SD) were then summed within participant to create the clustered score at years 7, 10, 15, and 20. Means and SD of year 7 were used for standardization at each following exam year.

#### 2.2.3. Hostility

Participants rated their levels of hostility by using the Cook-Medley hostility questionnaire at year 5 exam [19]. This survey reflects a participant's feelings of mistrust, anger, suspicion, and aggression. This questionnaire shows good convergent and discriminate validity [19]. A sample based median split was used to define high and low hostility groups, consistent with previously published approaches [20].

#### 2.2.4. Covariates

All covariates were assessed by interviewer-administered questionnaire at each CARDIA examination with the exception of diet. The continuous physical activity score was measured by intensity level and the number of months spent in 13 different activities of heavy (≥5 metabolic equivalents (METS)) and moderate (3-4 METS) intensity during the past year [21]. Diet was assessed at years 0, 7, and 20 using the CARDIA Diet History questionnaire [22]. The continuous dietary pattern score was assessed by types and amounts of food consumed over the past month. Foods were assigned into 46 groups which, in turn, were categorized as beneficial (*N* = 20), adverse (*N* = 13), and neutral (*N* = 13) [23]. Assessment of diet at year 7 was a surrogate for year 5 of this study.

## 3. Statistical Analyses

The Chi-square test was used to assess the significance of bivariate associations for categorical outcomes. One-way ANOVA tests were used to assess differences between subgroups for continuous outcomes. Wilcoxon signed-rank tests were used to examine the median difference on variables measured at year 20 and year 5 (e.g., hours of daily TV viewing). Structural equation modeling (SEM) in MPlus version 6 was used to run the cross-lagged panel models. A cross-lagged panel model was specified to examine the prospective relationships between TV viewing and cardiometabolic risk variables over a total of three five-year intervals. Cross-lagged panel models in a multiple group analysis were specified to examine hostile personality trait (i.e., high and low hostility groups) as a modifier of the association between TV viewing and cardiometabolic risk variables.

The cross-lagged panel models were adjusted for stable variables at baseline and time-varying variables at each exam year. Each cross-lagged panel model included autoregressive associations within the same variables, cross-lagged association for TV viewing and cardiometabolic risk variables to prospectively predict each other, and adjustment for covariates. A Chi-square test was used to evaluate model fit by computing the ratio of the two log-likelihoods from the observed and model-implied covariance matrices. Because the Chi-square test is sensitive to sample size, several other goodness of fit measures were used in the SEM analyses to assess model fit. Comparative Fit Index (CFI) ≥ 0.95, Tucker-Lewis Index (TLI) ≥ 0.95, and root mean squared error of approximation (RMSEA) ≤ 0.06 [24]. Modification indices were used to identify constrained or missing associations that if unconstrained or included, would improve fit.

## 4. Results

### 4.1. Participant Characteristics

Table 1 shows the demographic and behavioral characteristics of participants by hostility level at baseline (groups are not equal due to “ties”). The high hostility group reported lower mean age: 29.4 ± 3.7 years versus 30.4 ± 3.5 years for people with low hostility. People with high hostility had lower diet score (mean = 63.8 for the high hostility group; 69.6 for the low hostility group) but higher physical activity score (mean = 400.4 for the high hostility group; 377.2 for the low hostility group). Compared with the low hostility group, the high hostility group had more males (41.7% versus 58.3%) and Blacks (32.6% versus 67.4%). People who had less education than high school were more likely to be hostile (27.2% for the low hostility group versus 72.8% for the high hostility group). Among those whose annual family income was < 24,999, 63% were in the high hostility group versus 37% in the low hostility group.

### 4.2. Descriptive Analysis

As Table 2 shows, the high hostility group reported higher levels of TV viewing time, clustered cardiometabolic risk score, waist circumference, fasting glucose, insulin, triglycerides, and systolic blood pressure and lower values of HDL-cholesterol at each examination year. The high hostility group showed higher physical activity score at year 5 and 10, but lower physical activity score at year 15 and 20 compared with the low hostility group.

### 4.3. Correlations

As shown in Table 3, there were significant positive correlations between TV viewing and clustered cardiometabolic risk score, waist circumference, HOMA insulin resistance, and systolic blood pressure for both hostility groups. No significant correlations were observed between TV viewing and triglycerides and HDL-cholesterol for the high hostility group. There was a significant association between TV viewing at year 10 to triglycerides and HDL-cholesterol at 15 and at TV viewing at year 15 to triglycerides and HDL-cholesterol at year 20. The magnitude of the correlations tended to be small, as would be expected given measurement errors and the complicated etiology of the outcomes (i.e., TV exposure being only one of myriad determinants and risk factors).

### 4.4. Cross-Lagged Panel Model

**Figure 1:**
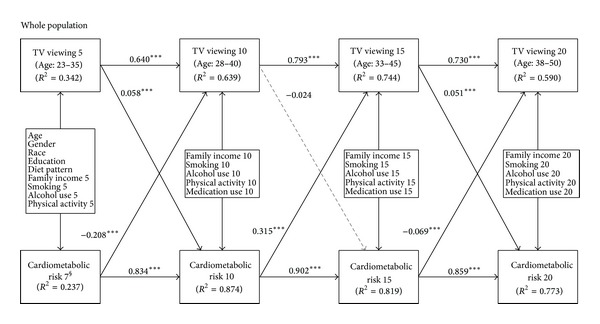
A cross-lagged panel model showing that TV viewing predicts increases in clustered cardiometabolic risk score for the whole population. Regression weights are standardized. *R* ^2^ represents the estimated proportion of the assumed underlying continuous variable explained by the model. § Cardiometabolic risk 7 is a surrogate of cardiometabolic risk 5. *P < 0.05; **P < 0.01; ***P < 0.001.

The results for the model summarized in Figure 1 supported the prospective association from TV viewing to cardiometabolic risk assumption and fit the data well, *χ* ^2^ (381) = 3566.31; CFI = 0.962; TLI = 0.950; RMSEA = 0.051. TV viewing exhibited significant temporal stability (*B* = 0.640, 0.793, and 0.730), as did clustered cardiometabolic risk score (*B* = 0.834, 0.902, and 0.859). In addition, the five-year lagged effect of TV viewing on clustered cardiometabolic risk score was significant and positive (*B* = 0.058 and 0.051) except for TV viewing at year 10 to clustered cardiometabolic risk score at year 15. Because these models control the autoregressive path, these 0.058 and 0.051 SD unit associations can be interpreted as prospective “increases” in the cardiometabolic risk score above the level of the risk score at the previous wave. The five-year lagged effect of clustered cardiometabolic risk score on TV viewing was significant, while the direction was inconsistent, being negative, positive, and negative over time (*B* = −0.208, 0.315, and −0.069).

### 4.5. Multiple Group SEM Analysis

**Figure 2:**
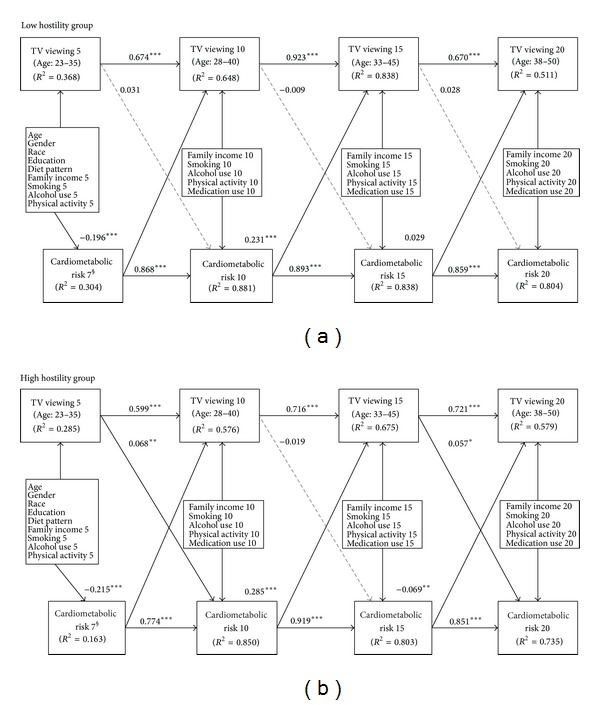
Two cross-lagged panel models showing the associations between duration of TV viewing and cardiometabolic risk are stronger for people with high hostility relative to those with low levels of hostility. Regression weights are standardized. *R* ^2^ represents the estimated proportion of the assumed underlying continuous variable explained by the model. § Cardiometabolic risk 7 is a surrogate of cardiometabolic risk 5. *P < 0.05; **P < 0.01; ***P < 0.001.

The results for the model summarized in Figure 2 supported the modifier assumption and fit the data well; CFI = 0.967; TLI = 0.957; RMSEA = 0.046, for the low hostility group; CFI = 0.970; TLI = 0.961; RMSEA = 0.050, for the high hostility group. For both groups, TV viewing exhibited significant temporal stability (*B* = 0.674, 0.923, and 0.670 for the low hostility group; *B* = 0.599, 0.716, and 0.721 for high hostility group), as did clustered cardiometabolic risk score (*B* = 0.868, 0.893, and 0.859 for the low hostility group; *B* = 0.774, 0.919, and 0.851 for the high hostility group). The five-year lagged effect of TV viewing on clustered cardiometabolic risk score was significant for those with high hostility (*B* = 0.068 for exam years 5 to 10 and 0.057 for exam years 15 to 20), whereas the effect was nonsignificant for those with low hostility.

Table 4 reports chi-square tests for difference testing between the low and high hostility groups, assessing whether clustered and individual associations are significantly different between these two cross-lagged panel models by hostility. A significant difference between baseline and structural invariance meant that these two overall models were significantly different (*χ* ^2^ (109) = 450.91; *P* < 0.001). The results of difference between structural invariance and partial structural invariance showed that all clustered and individual cross-lagged association of TV viewing and clustered cardiometabolic risk score were significantly different except the association of clustered cardiometabolic risk score at year 15 on TV viewing at year 20.

### 4.6. Cross-Lagged Models of Five Cardiometabolic Risk Variables

Table 5 represents the differential cross-lagged association from TV viewing to five cardiometabolic risk variables by hostility group from young to middle adulthood. There was no association between TV viewing and waist circumference for both groups. TV viewing time was positively associated with HOMA insulin resistance and systolic blood pressure for both groups. The coefficients linking TV viewing to HOMA insulin resistance and systolic blood pressure were higher in the high hostility group than the low hostility group. The association between TV viewing and HDL-cholesterol was inconsistent. TV viewing at year 5 for the low hostility group and at year 10 for the high hostility group was negatively related to triglycerides.

## 5. Discussion

Over 15 years of followup of a cohort of American adults from young to middle adulthood, there was a significant prospective association between hours of daily TV viewing and cardiometabolic risk. In particular, higher levels of TV viewing predicted an increase in clustered cardiometabolic risk score in adults from the ages of 23–35 to 28–40 and 33–45 to 38–50. Additionally, as hypothesized, there was an association between TV viewing and cardiometabolic risk was modified by hostility. As predicted, higher levels of TV viewing predicted an increase in clustered cardiometabolic risk score for people with high hostility but not for those with low levels of hostility, after controlling for potential confounding variables. The associations between TV viewing and HOMA insulin resistance and systolic blood pressure were stronger for those with high hostility relative to those with low hostility. Our study suggests that the association between TV viewing and cardiometabolic risk is stronger for those with high hostility relative to those with low levels of hostility. To our knowledge, this is the first study to assess the important hypothesis that the association between TV viewing and cardiometabolic risk may be modified by a propensity towards a hostile disposition.

The positive association between TV viewing and cardiometabolic risk was also observed in several cross-sectional studies [5, 6, 8, 25, 26] and two longitudinal studies [9, 10] in adults. This study indicated that the association between TV viewing and clustered cardiometabolic risk was independent of physical activity and dietary quality. Another mechanism that is possible for this association is through a relative decrease in energy expenditure from increased TV watching. Metabolic rate is lower during TV watching than during other sedentary behaviors including sewing, playing board games, and reading [27], and TV viewing is a stronger predictor of obesity relative to other sedentary behaviors [28]. Our findings that psychological attributes could be another potential mechanism through which TV viewing increases the likelihood of cardiometabolic risk add to this existing work. Two findings could support this hypothesis. The prospective association between TV viewing and clustered cardiometabolic risk score was found in hostile people who are more likely to have negative emotions induced by TV viewing. Additionally, the association between TV viewing and systolic blood pressure was stronger for those with high hostility compared with those with low hostility.

Epidemiological studies have supported the notion that consequences from TV viewing may be modified by hostility and aggression [29–33]. Hostility is generally believed to be a personality trait which often exists with anger, cynicism, and aggressive response [12, 19]. Among experimental studies, children were more likely to behave in a hostile way during social interaction after watching violent programs [34–36]. Among longitudinal studies, Johnson and colleagues found that watching TV during adolescence and young adulthood increased the likelihood of subsequent threatening aggression and assaults or fights [32]. Psychological theories propose explanations for the association between TV viewing and increased risk of both short- and long-term hostility and aggression [37–40]. Social cognitive theory suggests that children often imitate behaviors from the TV programs they watch, and thus they are more likely to become aggressive and violent due to excessive media violence exposure [41, 42]. Excitation transfer theory states that media violence and high risk activities increase psychological arousal, which causes subsequent hostile feelings and behaviors [42–44]. In addition, TV violence may desensitize an individual to cruel and violent scenes [45, 46].

Numerous studies have indicated that hostility may exacerbate cardiometabolic risks such as fasting glucose and blood pressure [47–50]. A meta-analysis of 25 studies suggested a positive effect of anger and hostility on coronary heart disease (hazard ratio [HR], 1.19; 95% confidence interval, 1.05 to 1.35). It has also been shown that hostility is predictive of cardiovascular risk factors [51]. Hostile people have a tendency to feel anger from frustrating situations, raising the possibility that images and messages on TV may promote stronger psychological responses and subsequent cardiometabolic risk for hostile people than for agreeable counterparts. Our findings supported the hypothesis derived from the finding that people with high hostility show a stronger association between TV viewing and clustered cardiometabolic risk score, HOMA insulin resistance, and systolic blood pressure relative to those with low levels of hostility.

The somewhat unique pattern of cross-lagged association over time deserves noting. The associations from TV to CMR are positive, negative (although not significantly so), and positive from year 5 to 10, 10 to 15, and 15 to 20, respectively, and the association from CMR to TV is negative, positive, and negative from year 5 to 10, 10 to 15, and 15 to 20, respectively. These specific patterns could produce cascading associations—higher TV viewing at year 5 increases CMR at year 10 which increases TV viewing at year 15 which increases CMR at year 20. Alternately, high CMR at year 7 decreases TV viewing at year 10 which increases CMR at year 15 which decreases TV viewing at year 20. However, since these are average group associations as opposed to individual trajectories, this specific pattern may not be produced at the individual level. Also, these associations occur against the backdrop of much stronger positive autoregressive correlations that produce consistent (as opposed to changing) levels of TV and CMR over time.

There are several limitations in our study. Due to the lack of TV content data, it is not possible to not assess what kind of TV program may evoke negative emotional reactions. The replacement of cardiometabolic risk at year 5 by year 7 could underestimate the association because of more follow-up missing data over time. It is possible that measurement error occurred due to the self-reported behavioral variables including hours of TV viewing. Our population only included the Black and White adults, so the results cannot be generalized to other populations. Additionally, the SEM analytical approach is powerful and takes into account fixed and random effects and time lag estimates, but the results are sometimes challenging to interpret compared to more traditional approaches.

This is the first study to explore the prospective association between TV viewing and cardiometabolic risk modified by a psychological factor. Our findings are of public health significance given that TV viewing and cardiometabolic risk are highly prevalent in the world. Future studies are needed to assess whether negative psychological effect is a potential mechanism mediating the relationship of TV viewing on cardiometabolic risk. Results of these studies may be helpful in the prevention of cardiovascular disease, possibly by tailoring interventions to reduce hostility and reducing hours of daily TV viewing in this subgroup, which may result in lower cardiometabolic risk.

## Figures and Tables

**Table 1:** Demographic and behavioral distribution of participants by levels of hostility at baseline (Year 5), CARDIA, 1985.

Characteristic	Low hostility	High hostility	*P* value	Total (*N* = 3269)
	(*N* = 1547)	(*N* = 1722)		*N* (%)
Age (y)	30.4 ± 3.5^a^	29.4 ± 3.7	<0.001^c^	29.9 ± 3.6
A priori diet quality score	69.6 ± 11.7	63.8 ± 12	<0.001^c^	66.6 ± 12.2
Physical activity score	377.2 ± 280.8	400.4 ± 310.8	0.03^c^	389.4 ± 297.2
Sex				
Male	660 (41.7)^b^	922 (58.3)	<0.001^d^	1582 (48.4)
Female	887 (52.6)	800 (47.4)		1687 (51.6)
Race				
Black	514 (32.6)	1062 (67.4)	<0.001^d^	1576 (48.2)
White	1033 (61.0)	660 (39.0)		1693 (51.8)
Highest education				
≤12 years	168 (27.2)	450 (72.8)	<0.001^d^	618 (18.9)
>12 years	1379 (52.0)	1272 (48.0)		2651 (81.1)
Family income (year)				
<24,999	471 (37.4)	788 (62.6)	<0.001^d^	1259 (38.5)
25,000–49,999	577 (48.2)	621 (51.8)		1198 (36.7)
≥50,000	499 (61.5)	313 (38.6)		812 (24.8)
Alcohol use (drinks/week)				
0	731 (50.5)	717 (49.5)	<0.001^d^	1866 (57.1)
1–6	562 (50.6)	549 (49.4)		1403 (42.9)
≥7	254 (35.8)	456 (64.2)		
Smoking status				1448 (44.3)
Never	975 (52.3)	891 (47.8)	<0.001^d^	1111 (34.0)
Former/current	572 (40.8)	831 (59.2)		710 (21.7)

^a^Mean ± SD (all such values).

^b^ *N* (%) (all such values).

^c^Results were tested by one-way ANOVA analysis.

^d^Results were tested by *χ* ^2^ test.

**Table 2:** Distribution of TV viewing, cardiometabolic risk variables, and physical activity score by hostility at examination year, CARDIA, 1985–2005.

Variables	Year 5	Year 10	Year 15	Year 20	Year 5 versus year 20
	Mean ± SD	Mean ± SD	Mean ± SD	Mean ± SD	*P* value^b^
Low hostility					
Exposure					
Hours of TV viewing daily	1.9 ± 1.6	1.9 ± 1.5	1.8 ± 1.5	1.9 ± 1.6	0.60
Outcome					
Clustered cardiometabolic risk score	−0.4 ± 3.4^a^	0.04 ± 3.5	0.9 ± 3.8	1.6 ± 4.0	<0.001
Waist circumference (cm)	81.9 ± 13.2	83.6 ± 13.6	87.6 ± 15.2	90.1 ± 14.9	<0.001
Fasting glucose (ug/dL)	88.2 ± 10.2^a^	86.1 ± 10.7	84.6 ± 14.2	95.1 ± 17.7	<0.001
Fasting insulin (uU/mL)	12.6 ± 7.7^a^	12.8 ± 9.5	13.5 ± 9.7	15.2 ± 9.5	<0.001
Fasting triglycerides (mg/dL)	80.4 ± 76.7	85.1 ± 57.9	101.2 ± 92.3	105.9 ± 79.4	<0.001
HDL-cholesterol (mg/dL)	51.9 ± 13.4	50.4 ± 13.2	51 ± 13.1	54.9 ± 16.0	0.008
Systolic blood pressure (mmHg)	106.3 ± 10.5	107.8 ± 10.9	110.2 ± 12.7	113.7 ± 13.9	<0.001
Covariate					
Physical activity score	377.8 ± 280.8	340.4 ± 260.3	359.2 ± 284.9	365.9 ± 285.8	0.03

High hostility					
Exposure					
Hours of TV viewing daily	3 ± 2.4	2.8 ± 2.3	2.7 ± 2.2	2.9 ± 2.7	0.15
Outcome					
Clustered cardiometabolic risk score	0.4 ± 3.5^a^	0.9 ± 3.6	1.7 ± 3.8	2.5 ± 3.9	<0.001
Waist circumference (cm)	85 ± 13.9	86.9 ± 14.8	90.6 ± 15.3	93 ± 15.5	<0.001
Fasting glucose (ug/dL)	89.9 ± 15.8^a^	88.2 ± 16.5	86.4 ± 18.4	99.2 ± 28.6	<0.001
Fasting insulin (uU/mL)	14.5 ± 12.1^a^	14.2 ± 9.5	14.7 ± 11.6	16.7 ± 11.4	<0.001
Fasting triglycerides (mg/dL)	83.7 ± 63.8	92.4 ± 80.4	102.7 ± 76.2	109.2 ± 76.2	<0.001
HDL-cholesterol (mg/dL)	51.6 ± 14.3	50 ± 14.6	49.9 ± 14.6	53 ± 16.7	0.02
Systolic blood pressure (mmHg)	108.9 ± 11.2	110.2 ± 12.2	114.1 ± 14.4	117.2 ± 14.8	<0.001
Covariate					
Physical activity score	400.4 ± 310.8	348.5 ± 295	358.2 ± 291.7	340.7 ± 277.2	<0.001

^a^Results were assessed at year 7 but not at year 5.

^b^Results were tested by Wilcoxon signed-rank test for the median difference between year 20 and year 5.

**Table 3:** Correlations between TV viewing and cardiometabolic risk variables by hostility, CARDIA, 1985–2005.

Low hostility	Clustered 10	WST 10	HOMA 10^b^	TRI 10^b^	HDL 10	SBP 10
TV viewing 5						
r^a^	0.14∗∗∗	0.16∗∗∗	0.19∗∗∗	0.02	−0.04	0.11∗∗∗
*P* value	<0.001	<0.001	<0.001	0.39	0.17	<0.001

Low hostility	Clustered 15	WST 15	HOMA 15^b^	TRI 15^b^	HDL 15	SBP 15

TV viewing 10						
r	0.22∗∗∗	0.21∗∗∗	0.22∗∗∗	0.08∗∗	−0.08∗∗	0.16∗∗∗
*P* value	<0.001	<0.001	<0.001	0.008	0.009	<0.001

Low hostility	Clustered 20	WST 20	HOMA 20^b^	TRI 20^b^	HDL 20	SBP 20

TV viewing 15						
r	0.22∗∗∗	0.23∗∗∗	0.21∗∗∗	0.06∗	−0.11∗∗∗	0.16∗∗∗
*P* value	<0.001	<0.001	<0.001	0.05	<0.001	<0.001

High hostility	Clustered 10	WST 10	HOMA 10^b^	TRI 10^b^	HDL 10	SBP 10

TV viewing 5						
r	0.12∗∗∗	0.12∗∗∗	0.14∗∗∗	0.03	0.02	0.11∗∗∗
*P* value	<0.001	<0.001	<0.001	0.19	0.46	<0.001

High hostility	Clustered 15	WST 15	HOMA 15^b^	TRI 15^b^	HDL 15	SBP 15

TV viewing 10						
r	0.14∗∗∗	0.12∗∗∗	0.14∗∗∗	0.02	−0.01	0.15∗∗∗
*P* value	<0.001	<0.001	<0.001	0.57	0.67	<0.001

High hostility	Clustered 20	WST 20	HOMA 20^b^	TRI 20^b^	HDL 20	SBP 20

TV viewing 15						
r	0.16∗∗∗	0.12∗∗∗	0.13∗∗∗	0.05	−0.02	0.20∗∗∗
*P* value	<0.001	<0.001	<0.001	0.08	0.45	<0.001

Clustered: clustered cardiometabolic risk; WST: waist circumference; HOMA: HOMA insulin resistance; TRI: triglycerides; HDL: HDL-cholesterol; SBP: systolic blood pressure.

^a^Results were tested by Spearman's rank correlation test.

^b^Variables were log-transformed (natural log).

*P < 0.05;  **P < 0.01;  ***P < 0.001.

**Table 4:** Chi-square tests for difference testing between the low and high hostility groups, CARDIA, 1990–2005.

Chi-square test for difference testing	Value	df^a^	*P* value
Baseline versus structural invariance	450.91	109	<0.001
Structural invariance versus partial structural invariance			
6 cross-lagged associations	27.99	6	<0.001
3 associations of TV on cardiometabolic risk	21.26	3	<0.001
TV 5 → cardiometabolic risk 10	8.72	1	0.003
TV 10 → cardiometabolic risk 15	15.21	1	<0.001
TV 15 → cardiometabolic risk 20	8.99	1	0.003
Cardiometabolic risk 7 → TV 10	4.84	1	0.03
Cardiometabolic risk 10 → TV 15	6.9	1	0.009
Cardiometabolic risk 15 → TV 20	1.37	1	0.24

^a^df: degree of freedom.

**Table 5:** Cross-lagged associations from TV viewing to five cardiometabolic risk variables by levels of hostility.

Low hostility	WST 10	HOMA 10^a^	TRI 10^a^	HDL 10	SBP 10
TV viewing 5					
*B *	0.009	0.046∗	−0.079∗∗	0.075∗∗	0.119∗∗∗
*P* value	0.63	0.03	0.005	0.002	<0.001

Low hostility	WST 15	HOMA 15^a^	TRI 15^ a^	HDL 15	SBP 15

TV viewing 10					
*B *	0.025	0.055∗∗	−0.051	0.000	0.037
*P* value	0.14	0.009	0.08	0.99	0.10

Low hostility	WST 20	HOMA 20^a^	TRI 20^a^	HDL 20	SBP 20

TV viewing 15					
*B *	0.005	0.024	−0.008	−0.056∗∗	0.053∗
*P* value	0.75	0.25	0.78	0.006	0.02

High hostility	WST 10	HOMA 10^a^	TRI 10^a^	HDL 10	SBP 10

TV viewing 5					
*B *	0.035	0.064∗∗	−0.02	0.065∗∗	0.177∗∗∗
*P* value	0.17	0.001	0.48	0.001	<0.001

High hostility	WST 15	HOMA 15^a^	TRI 15^a^	HDL 15	SBP 15

TV viewing 10					
*B *	−0.032	0.002	−0.064∗	−0.086∗∗∗	−0.006
*P* value	0.09	0.95	0.03	<0.001	0.85

High hostility	WST 20	HOMA 20^a^	TRI 20^a^	HDL 20	SBP 20

TV viewing 15					
*B *	−0.006	0.04	0.02	−0.034	0.160∗∗∗
*P* value	0.75	0.68	0.51	0.07	<0.001

WST: waist circumference; HOMA: HOMA insulin resistance; TRI: triglycerides; HDL: HDL-cholesterol; SBP: systolic blood pressure. All cross-lagged panel models were adjusted for the same covariates as the clustered cardiometabolic risk model. Regression weights are standardized.

^a^Variables were log-transformed (natural log).

*P < 0.05;  **P < 0.01;  ***P < 0.001.
